# Risk factors for renal dysfunction after total hip joint replacement; a retrospective cohort study

**DOI:** 10.1186/s13018-015-0299-0

**Published:** 2015-10-01

**Authors:** Basim Kamil Hassan, Arne Sahlström, Ram Benny Christian Dessau

**Affiliations:** Department of Orthopedics, Nykoebing Falster Hospital, Fjordvej 15, 4800 Nykoebing Falster, Denmark; Department of Clinical Microbiology, Slagelse Hospital, Ingemannsvej 18, 4200 Slagelse, Denmark

## Abstract

**Background and purpose of the study:**

Renal injury and dysfunction are serious complications after major surgery, which may lead to increased morbidity and mortality. The objective of our study was to identify the possible risk factors for renal dysfunction after total hip joint replacement surgery.

**Methods:**

A retrospective study was conducted among 599 consecutive primary hip joint replacements performed between January 2011 and December 2013. According to the RIFLE criteria, increased postoperative serum creatinine was considered indicative of postoperative renal injury. The Welch two-sample test, chi-square test, and Fisher exact test were used for statistical analysis.

**Results:**

Eighty-one patients (13.8 %) had significant moderate or severe postoperative renal dysfunction in which 10 patients (1.7 %) acquired severe and permanent renal impairment.

**Conclusion:**

We identified advanced age, hypertension, general anesthesia, high ASA scores, low intra-operative systolic BP, and prophylactic dicloxacillin as significant risk factors. Low baseline systolic BP, low baseline diastolic blood pressure, and hip fracture diagnosis were independent risk factors for postoperative increase in serum creatinine. Smoking, diabetes mellitus, high BMI, gender, and duration of surgery were not identified as significant risk factors.

## Introduction

Total hip joint replacement is indicated mainly for hip osteoarthritis, for complications after osteosynthesis of hip fractures, and for the treatment of femoral neck fractures in relatively young patients. Possible complications are deep venous thrombosis [1–3], infection [4–6], dislocation of the hip prosthesis [7, 8] and increased creatinine levels, and impaired renal function [9–11]; the latter may in turn increase mortality and morbidity among patients who are already affected by diseases such as diabetes mellitus, hypertension, heart disease, and obesity [12–16]. The aim of this study was to identify patients with renal injury after total hip joint replacement and to detect possible risk factors and their clinical relevance in our retrospective material of 599 consecutive total hip joint replacements. In recent years, a few studies identified renal impairment as a complication to be considered after major surgery [17–21].

## Materials and methods

A retrospective study was performed which included a consecutive cohort of patients who underwent primary total hip joint replacement using cementless CORAIL®stem with either Pinnacle or Avantage cup, between January 2011 and December 2013. Indications for surgery were primary osteoarthritis (*n* = 551), femoral neck fractures, and complications after osteosynthesis of hip fractures (*n* = 48). A total of 599 patients with a total of 599 hip joint replacements were included. Data was obtained from our computerized database and hospital charts. Charts were reviewed for at least 9 months after surgery. Out of the 599 total hip joint replacements, 588 had complete data sets matching our investigation criteria. The following variables were selected [17, 18]: age, sex, body mass index (BMI), hypertension, diabetes mellitus, smoking, American Society of Anesthesiologists (ASA) physical status, prophylactic antibiotics according to our protocol (one dose immediately preoperatively and three doses in the first postoperative day), duration of surgery, type of anesthesia, baseline systolic blood pressure (BP), baseline diastolic BP, intra-operative systolic BP, and intra-operative diastolic BP (lowest measured blood pressure intra-operatively).

Furthermore, 11 patients were not included due to the missing intra-operative BP data. Two patients were excluded due to pre-existing severe renal dysfunction (in hemodialysis) because any new renal injury could not have been detected. Five hundred eighty-six patients, with complete data set and inclusion criteria, were available for analysis.

In our department, the protocol for elective total hip joint replacement surgery includes measuring serum creatinine; once preoperatively and three consecutive days postoperatively. Increased postoperative serum creatinine was monitored and controlled daily until it decreased or the patient was referred to the nephrology department. During the first postoperative week, the highest serum creatinine was chosen as a sign for maximum renal injury. Dicloxacillin was the antibiotic of choice for prophylaxis and cefuroxime used as the alternative in cases of allergies to penicillin.

Patients were identified as renally impaired using the relative increase in serum creatinine and the RIFLE classification proposed by the Acute Dialysis Quality Initiative Group to identify patients with renal impairment [19–21]. The patients were accordingly divided into two groups, those with RIFLE < 1.5 times increase in serum creatinine where renal impairment is absent or mild and those with RIFLE ≥ 1.5 times increase in serum creatinine indicating moderate or severe renal impairment (Table 1).

**Table 1:** The RIFLE classification

	GFR criteria	Urine output criteria
Risk	SCr increased 1.5 times	0.5 ml(kg h) for 6 h
Injury	SCr increased 2.0 times	0.5 ml(kg h) for 12 h
Failure	SCr increased 3.0 times or creatinine = 355 μmol/l when there was an acute rise of >44 μmol/l	0.3 ml(kg h) for 24 h or anuria for 12 h
Loss	Persistent ARF; complete loss of kidney function for >4 weeks	
End-stage renal disease	End-stage renal disease for >3 months	

For statistical analysis and graphics, the free R software was used (www.r-project.org). Multivariate regression analysis was performed using the command generalized linear model. Model reduction was performed objectively using an automated procedure (step) maximizing Akaike’s information criterion (AIC). For the multivariate regression model, the relative increase in serum creatinine was used as a continuous variable. Univariate comparisons were made using the Welch two-sample test, chi-square test, and Fishers exact test. A *P* value of 0.05 or less was considered significant.

The study has been approved by the Danish Data Management Board, and it has been conducted in accordance with the ethical and legal requirements of the Institutional Review Board of Sjaelland region.

## Results

**Fig. 1:**
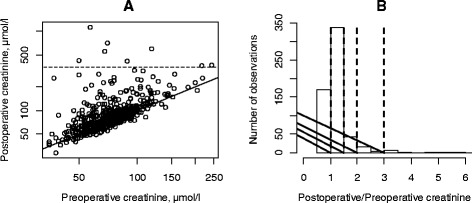
**a** XY plot of preoperative versus postoperative serum creatinine. The patients had a mean increase in postoperative serum creatinine of 8 μmol/l (0.0–15.4, 95 % confidence interval on the difference, *P* = 0.05 paired *t*-test). The *diagonal line* depicts no change. The *broken line* is set at the limit of 355 μmol/l (see Table 2). The normal range for women is 50–90 μmol/l and for men 60–105 μmol/l. **b** Histogram of relative change in serum creatinine. The mean relative change was 1.2. The *vertical broken lines* depict 1 = no change, 1.5, 2, and 3 according to the RIFLE classification

During the study, 81 out of 586 patients had significant moderate or severe renal impairment (RIFLE ≥ 1.5) resulting in an overall incidence of 13.8 % (Table 2). Forty-six patients (7.8 %) had RIFLE 1.5–2, 19 patients (3.2 %) had RIFLE 2–3, and 16 patients (2.7 %) had RIFLE ≥ 3. Out of these 81 patients, 71 improved but 10 patients acquired severe and permanent renal impairment (i.e., in dialysis) with an incidence of 1.7 %. Seven patients had postoperative serum creatinine above the defined failure limit (355 μmol/l). This was not correlated with a higher preoperative serum creatinine (Fig. 1a). The two patients with high preoperative serum creatinine were already above 200 μmol/l. They had only a smaller relative increase in serum creatinine (Fig. 1a, b). The renal status of the 81 patients was observed through electronic charts for at least 9 months after surgery.

**Table 2:** The variables advanced age, general anesthesia, hypertensive disease, and high ASA scores revealed significant postoperative renal dysfunction. Patients not receiving dicloxacillin preoperatively were given cefuroxime

Variables	RIFLE < 1.5	RIFLE ≥ 1.5	*P* value	Test
	*n* = 505	*n* = 81		
Mean age	69 (range 37–93)	73 (range 49–91)	0.002*	T
Mean BMI	27.4 (range 15–46)	27.5 (range 18–42)	0.77	T
Duration of Surgery (minutes)	64 (range 30–223)	65 (range 30–161)	0.67	T
Baseline systolic BP	147 (range 90–206)	154 (range 115–231)	0.011	T
Baseline diastolic BP	83 (range 40–121)	80 (range 50–114)	0.05	T
Intra-operative systolic BP	90 (range 60–145)	89 (range 60–170)	0.53	T
Intra-operative diastolic BP	52 (range 30–90)	50 (range 35–80)	0.24	T
General anesthesia	265 yes/240 no	53 yes/28 no	0.04*	C
Gender	229 M/276 F	31 M/50 F	0.28	C
Smoking	386 no/119 yes	65 no/16 yes	0.53	C
Hypertensive patients	264	56	0.006*	C
Normotensive patients	241	25		
Diabetes mellitus	456 no/49 yes	73 no/8 yes	1	C
ASA score 1	91	5	0.006*	C
ASA score 2	319	52		
ASA score 3	95	24		
Dicloxacillin	53 no/452 yes	3 no/78 yes	0. 084	F

*T* Welch two sample test, *C* chi-square test, *F* Fisher exact test

**Table 3:** Model output after stepwise reduction. The dependent variable was the relative change in serum creatinine defined as postoperative creatinine/preoperative creatinine

	Estimate	Std. error	*P* value
Age	0.003509	0.001502	0.0198*
BMI	0.004841	0.002954	0.1018
Diabetes mellitus	−0.076695	0.046419	0.0990
Hypertension	0.043715	0.02879	0.1285
General anesthesia	0.072565	0.027415	0.0083*
Dicloxacillin	0.157739	0.046075	0.0007*
Baseline systolic BP	−0.003150	0.000755	0.0001*
Baseline diastolic BP	0.004641	0.001362	0.0007*
Diagnosis fracture	0.136877	0.051407	0.0079*

Table 2 reveals advanced age, hypertension, general anesthesia, high ASA scores, low intra-operative BP, and using prophylactic dicloxacillin as being significant risk factors for renal impairment, after total hip joint replacement on univariate analysis.

Generalized multivariate modeling was performed using the relative change in serum creatinine as a dependent variable. It confirmed that advanced age, hypertension, general anesthesia, prophylactic dicloxacillin, low baseline systolic and diastolic BP, and having a hip fracture diagnosis were significant independent risk factors for a rise in serum creatinine (Table 3).

BMI, duration of surgery, gender, diabetes mellitus, and smoking were not considered significant risk factors.

## Discussion

Increased hospital stay, morbidity, mortality, and increased cost may all be consequences of acute postoperative renal dysfunction [22, 23]. To date, preventative strategies are the only effective measures to reduce morbidity in cases of postoperative renal dysfunction. Therefore, in order to influence our guidelines, it is imperative to identify the risk factors of renal dysfunction after total hip joint replacement surgery.

In spite of the retrospective design, data was complete for most patients; only 11 patients were excluded from the study due to missing data. However, an important limitation was the missing information on fluid input and output which would have potential influence on renal function. Unfortunately, these charts were unreliable and had frequent missing records of blood loss during surgery. Therefore, data regarding perioperative blood loss was not collected. None of our patients had received blood transfusions perioperatively, and very few patients received blood transfusion postoperatively (<1 %) indicating minimal blood loss during surgery. Excessive blood loss during surgery may lead to decreased intra-operative BP and renal blood flow predisposing the patients to pre-renal failure. Our study shows that a higher preoperative serum creatinine is not a predictor for either a higher postoperative serum creatinine above the limit of 355 μmol/l or a higher relative change (Fig. 1a).

In accordance with Mantilla et al. [1], Parvizi et al. [3], Aveline et al. [9], Nergelius et al. [10], Abelha et al. [11], and Jämsen et al. [23], we found increased age as an independent risk factor for renal dysfunction after major surgery. However, Sharrock et al. [13] was not able to confirm the age factor in this regard. This may have been due to the relatively small number of patients included.

Our patients received either general anesthesia (*n* = 318) or spinal anesthesia (*n* = 268). General anesthesia was an independent risk factor for the development of postoperative renal dysfunction [24]. The type of anesthesia was chosen by the attending anesthesiologist only after an individual clinical assessment of each patient was performed. Thus, this observation may have been influenced by preferences of the anesthesiologist. Jafari et al. [17] did not report this finding—perhaps due to inadequate data regarding the number of patients who received general anesthesia or other forms of anesthesia.

Our patients received prophylactic antibiotics in the form of either dicloxacillin (*n* = 530) or cefuroxime (*n* = 56). Those receiving the former had a significant increased risk of increased postoperative serum creatinine. Baily et al. [25], Solgaard et al. [26], and Isacson and Collert [27] developed the same conclusion in their respective studies. Dicloxacillin has been the local recommendation for many years due to the narrow bacterial spectrum relevant to prevent infections with *Staphylococcus aureus*. In addition, dicloxacillin compared to cefuroxime is known to have a lower risk of complications concerning gastrointestinal problems and induction of bacterial resistance [28, 29].

The ASA score was an independent significant risk factor for the development of renal impairment, thus corresponding with the findings of Parvizi et al. [3], Abelha et al. [11], Belmont et al. [16], and Jafari et al. [17].

In our study, hypertensive disease (under treatment) had a significant increase in the risk for renal impairment as supported by Nergelius et al. [10], Naik et al. [21], and Weingarten et al. [24]. In addition, patients with low baseline systolic and diastolic BP, before anesthesia induction, also had an increased risk for renal impairment. This may be due to a reduced capacity to tolerate an additional drop in BP during anesthesia induction.

Several authors [3, 15–17, 24, 30] have indicated that high BMI was an independent risk factor after joint replacement surgery. Although our BMI range was 15 to 46, we could not confirm this finding.

Weingarten et al. [24] found that diabetes mellitus was independently associated with a high risk of developing acute kidney injury after total joint replacement, which was not the case in our study. However, Weingarten et al. [24] did not mention the actual diabetic disease control whereby our patients were meticulously controlled preoperatively.

Our study revealed a relatively high incidence of renal impairment (2.7 %) after primary total hip replacement compared to other studies [3, 17, 24]. The retrospective study conducted by Jafari et al. [17] showed an incidence of 0.55 % of acute renal failure or injury after joint arthroplasties (98 out of 17,938 joint arthroplasties including revision arthroplasties). Parvizi et al. [3] had an incidence of 0.85 % of acute renal failure in their prospective study of 1636 primary hip and knee joint replacements. The incidence was higher (1.82 %) in the retrospective study conducted by Weingarten et al. [24] which included a cohort of 9171 patients in which 167 patients showed acute kidney injury postoperatively. Nykoebing Falster Hospital serves an area of Denmark with a relatively older population and relatively low social status which would explain the higher risk of renal impairment.

Therefore, it is recommended that further studies be conducted and include controlled randomization to elucidate causal factors concerning postoperative renal impairment, after major surgery.

## Conclusion

Our study, in accordance with other studies, confirms the increased risk of renal injury after total hip joint replacement surgery. These findings may warrant a change in the protocol for informed consent as well as preoperative preparation protocols. Patients intended for total hip joint replacement may have to be informed preoperatively of any increased risk of renal impairment. High-risk patients (advanced age, hypertensive disease, and high ASA scores) should be indentified early for further optimization pre- and intra-operatively.
